# Markers of immune dysregulation in response to the ageing gut: insights from aged murine gut microbiota transplants

**DOI:** 10.1186/s12876-022-02613-2

**Published:** 2022-12-21

**Authors:** Panagiotis Giannos, Konstantinos Prokopidis, Masoud Isanejad, Helen L. Wright

**Affiliations:** 1 Society of Meta-Research and Biomedical Innovation, London, UK; 2 Department of Life Sciences, Faculty of Natural Sciences, Imperial College London, London, UK; 3 Department of Musculoskeletal and Ageing Science, Institute of Life Course and Medical Sciences, University of Liverpool, William Henry Duncan Building, 6 West Derby Street, Liverpool, L7 8TX UK

**Keywords:** Inflammation, Immune system, Gut microbiota, Ageing, Innate immunity, Differentially expressed genes

## Abstract

**Background:**

Perturbations in the composition and diversity of the gut microbiota are accompanied by a decline in immune homeostasis during ageing, characterized by chronic low-grade inflammation and enhanced innate immunity. Genetic insights into the interaction between age-related alterations in the gut microbiota and immune function remain largely unexplored.

**Methods:**

We investigated publicly available transcriptomic gut profiles of young germ-free mouse hosts transplanted with old donor gut microbiota to identify immune-associated differentially expressed genes (DEGs). Literature screening of the Gene Expression Omnibus and PubMed identified one murine (*Mus musculus*) gene expression dataset (GSE130026) that included small intestine tissues from young (5–6 weeks old) germ-free mice hosts that were compared following 8 weeks after transplantation with either old (~ 24-month old; *n* = 5) or young (5–6 weeks old; *n* = 4) mouse donor gut microbiota.

**Results:**

A total of 112 differentially expressed genes (DEGs) were identified and used to construct a gut network of encoded proteins, in which DEGs were functionally annotated as being involved in an immune process based on gene ontology. The association between the expression of immune-process DEGs and abundance of immune infiltrates from gene signatures in normal colorectal tissues was estimated from The Cancer Genome Atlas (TCGA) and Genotype-Tissue Expression (GTEx) project. The analysis revealed a 25-gene signature of immune-associated DEGs and their expression profile was positively correlated with naïve T-cell, effector memory T-cell, central memory T-cell, resident memory T-cell, exhausted T-cell, resting Treg T-cell, effector Treg T-cell and Th1-like colorectal gene signatures.

**Conclusions**

These genes may have a potential role as candidate markers of immune dysregulation during gut microbiota ageing. Moreover, these DEGs may provide insights into the altered immune response to microbiota in the ageing gut, including reduced antigen presentation and alterations in cytokine and chemokine production.

**Supplementary Information:**

The online version contains supplementary material available at 10.1186/s12876-022-02613-2.

## Background

Ageing is accompanied by a progressive decline in immune function that may significantly impact overall human health, increasing the risk of autoimmune disorders, infection, and mortality [1]. It has been proposed that immunosenescence in ageing populations may, in part, be driven by alterations in the composition and diversity of the gut microbiota, which confers a fundamental immunomodulatory role at promoting intestinal integrity and reducing local and systemic inflammation [2]. Particularly, aged gut microbiota can contribute to inflammageing, increased gut permeability, and enhanced bacterial leakage along with dysregulated innate immune responses [3–6] when transferred into young germ-free mice [7].

Current understanding of the interactions between age-related changes in the gut microbiota and immune function under a genetic perspective, remain poorly explored. To this end, we searched for publicly available transcriptomic gut profiles of young germ-free mouse hosts transplanted with old donor gut microbiota to identify immune-associated differentially expressed genes (DEGs). Identifying gene makers of immune dysregulation during gut microbiota ageing may contribute to mechanistic insights in preventing age-related alterations of intestinal physiology and associated disease.

## Methods

### Collection of microarray datasets

We screened the literature from inception until January 2022 by querying the National Center for Biotechnology Information (NCBI) Gene Expression Omnibus (GEO) using the search terms: (microbiome OR microbial OR microbiota OR microflora OR dysbiosis). We additionally searched the National Library of Medicine (NLM) PubMed using the additional terms: (differentially expressed genes OR DEGs). Two authors (PG and KP) created the search strategy and conducted the screening of the retrieved datasets.

Datasets were restricted based on organism type (*Mus musculus*), expression profiling (microarray), sample type (gastrointestinal tract), and condition (differential-microbiota conventionalization). Our search was free of any restrictions without exclusion criteria based on the baseline characteristics of murine models from which gut tissue samples were obtained. Datasets with no control samples were excluded from the search. Literature screening of the GEO and PubMed databases resulted in one expression dataset (GSE130026) on gut (small intestine) samples from young (5–6 weeks old) germ-free mouse hosts that were compared following 8 weeks after transplantation with either old (~ 24-month old; *n* = 5) or young (5–6 weeks old; *n* = 4) mouse donor gut microbiota [8].

### Identification of differentially expressed genes

Differentially expressed genes were retrieved using GEO2R according to the linear models for microarray analysis. DEGs following a *P* < 0.05 corrected by the Benjamini–Hochberg False Discovery Rate were considered as significant. Those with a positive log_2_ fold change (FC) were regarded as upregulated and with a negative log_2_FC as downregulated. This approach was ensued to amplify the coverage of all possible DEGs in gut microbiota ageing without overestimating the precision.

### Construction of protein–protein interaction network

Gut DEGs were used to construct a network of encoded proteins through The Search Tool for the Retrieval of Interacting Genes (STRING) [9]. Protein–protein interactions (PPI) in the network were approximated via a medium probabilistic confidence score > 0.4 and visualized with Cystoscope. The consideration of a reasonably moderate interaction cut-off was followed as to widen the coverage of potential protein interactions while attenuating their by-luck association.

### Immune-associated functional enrichment

DEGs were functionally enriched using gene ontology (GO) annotations with a probability density *P* < 0.05 following Benjamini–Hochberg False Discovery Rate correction, into GO terms of biological process (BP). DEGs highly enriched in immune-associated BP terms were obtained using the Molecular Signatures Database (MSigDB) [10, 11]. Ingenuity Pathway Analysis (IPA) was used to predict canonical pathways and upstream regulators that were activated or inhibited in response to DEGs [12].

### Immune cell infiltration prediction

The association between immune-associated DEGs and the microenvironment status in normal gut tissues via publicly available transcriptome data from The Cancer Genome Atlas (TCGA, (https://www.cancer.gov/tcga.) and Genotype-Tissue Expression (GTEx, https://gtexportal.org/home/) project, was investigated using the Gene Expression Profiling Interactive Analysis 2 (GEPIA2) [13]. Their expression with the abundance of immune infiltrates from gene signatures in normal colorectal tissues was estimated using Spearman’s correlation.

## Results

### Differentially expressed genes in old gut microbiota transplants

A total of 112 DEGs were obtained in the small intestine of young recipient mice transplanted with old donor gut microbiota when compared to age-matched young donor counterparts (Additional file 1, Additional file 2). Of these, 24 upregulated and 90 downregulated DEGs were identified.

### Protein interactome, immune-associated annotation and infiltration in old gut microbiota transplants

**Fig. 1:**
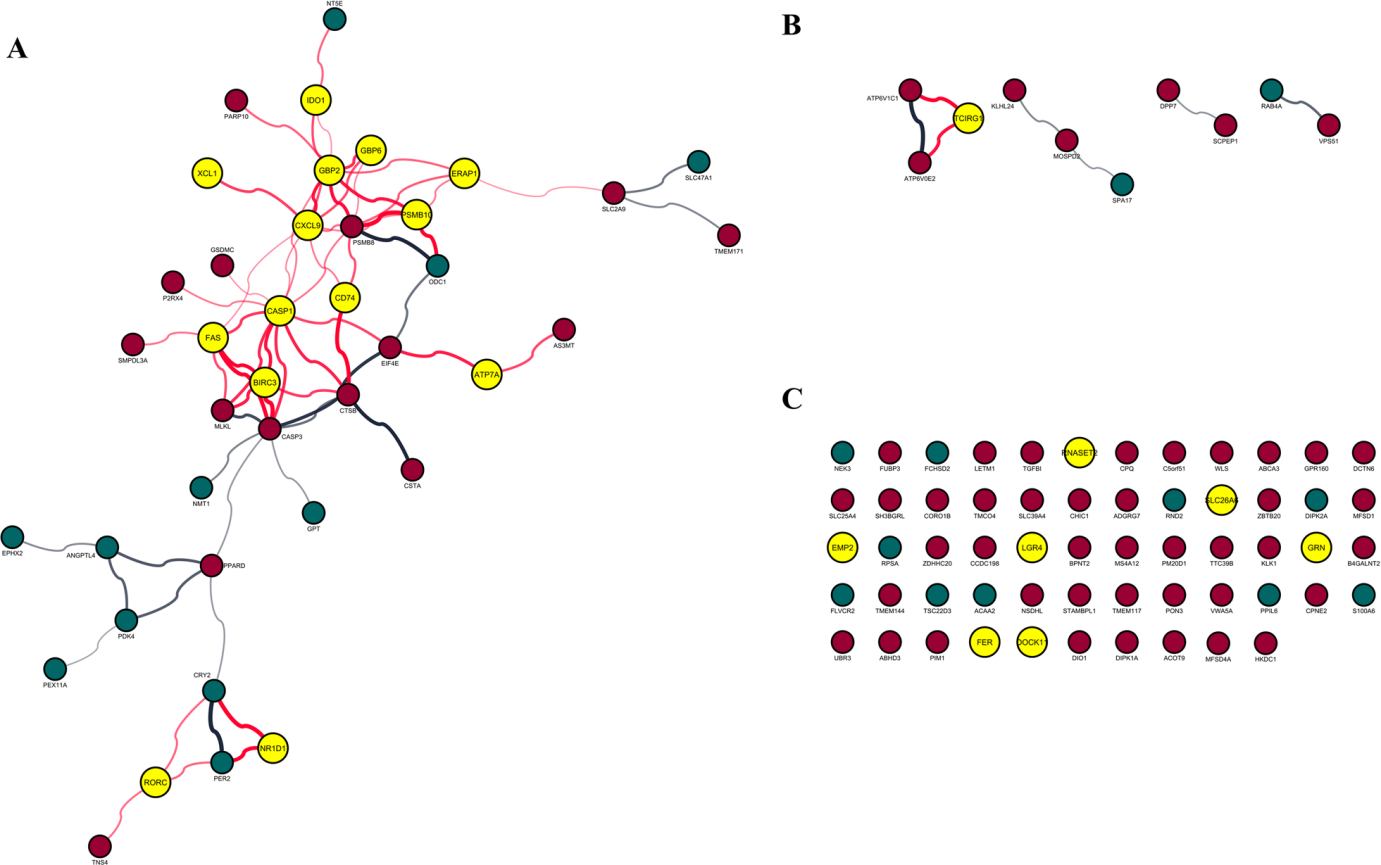
Network of encoded proteins of differentially expressed genes (DEGs) in the small intestine of young germ-free mouse hosts after transplantation with old donor gut microbiota. DEGs with multiple network interactions (**A**), few interactions (**B**) and no interactions (**C**). Immune-associated genes are shown as yellow, red represents upregulated genes and teal represents downregulated genes

**Fig. 2:**
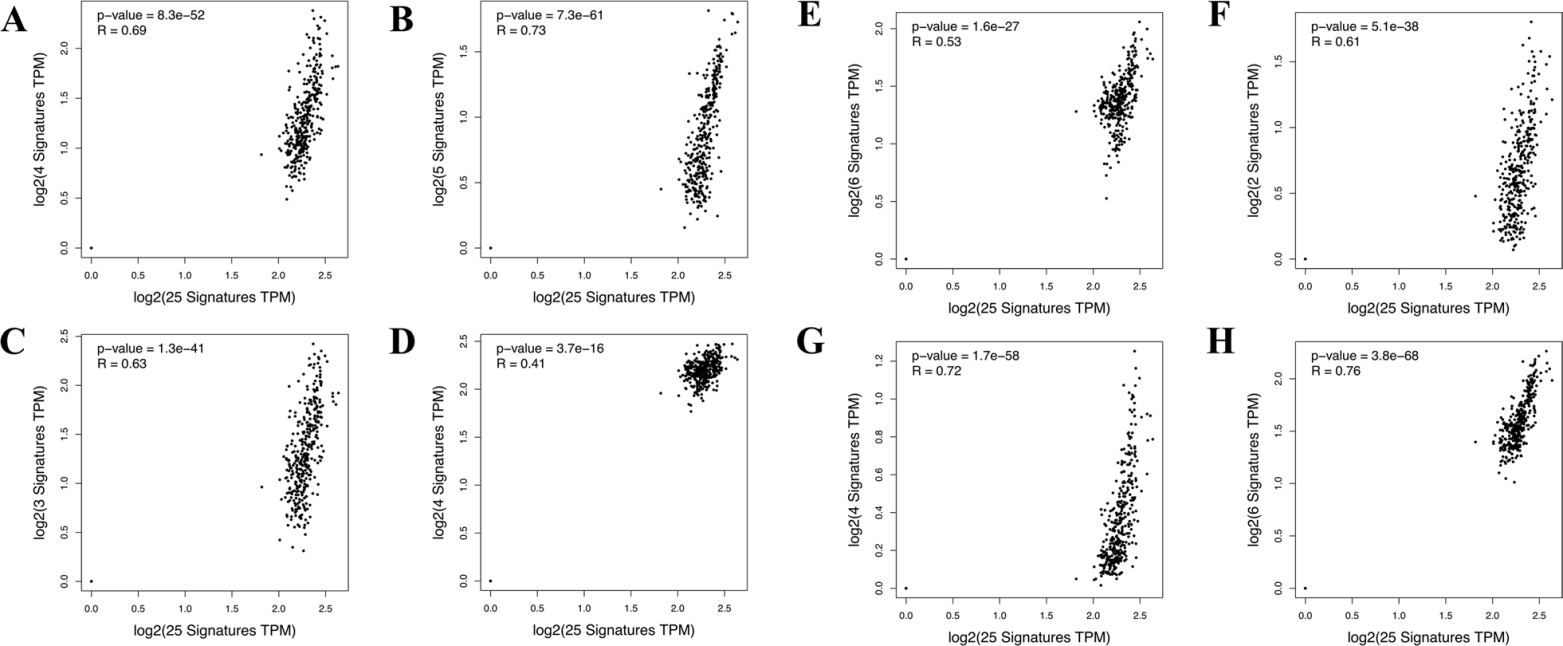
Immune microenvironment status of the 25-genes signature of immune-associated differentially expressed genes in the small intestine of young germ-free mice transplanted with old donor gut microbiota. Expression levels of this signature was positively correlated with naïve T-cell (**A**), effector memory T-cell (**B**), central memory T-cell (**C**), resident memory T-cell (**D**), exhausted T-cell (**E**), resting Treg T-cell (**F**), effector Treg T-cell (**G**) and Th1-like (**H**) gene signatures in normal colorectal tissues from The Cancer Genome Atlas and Genotype-Tissue Expression project

A network of 108 encoded proteins with 73 interactions of small intestinal DEGs from recipient mice transplanted with old gut microbiota, was constructed (Fig. 1). A 25-gene signature of DEGs was enriched in the GO Term “immune response” (GO:0006955, *P* = 6.51E−8): ATPase copper transporting alpha (ATP7A), baculoviral IAP repeat containing 3 (BIRC3), caspase 1 (CASP1), CD74 molecule (CD74), C-X-C motif chemokine ligand 9 (CXCL9), dedicator of cytokinesis 11 (DOCK11), epithelial membrane protein 2 (EMP2), endoplasmic reticulum aminopeptidase 1 (ERAP1), Fas cell surface death receptor (FAS), FER tyrosine kinase (FER), guanylate binding protein 2 (GBP2), guanylate binding protein family member 6 (GBP6), granulin precursor (GRN), major histocompatibility complex, class II, DM alpha (HLA-DMA), major histocompatibility complex, class II, DM beta (HLA-DMB), major histocompatibility complex, class II, DQ beta 2 (HLA-DQB2), indoleamine 2,3-dioxygenase 1 (IDO1), leucine rich repeat containing G protein-coupled receptor 4 (LGR4), nuclear receptor subfamily 1 group D member 1 (NR1D1), proteasome 20S subunit beta 10 (PSMB10), ribonuclease T2 (RNASET2), RAR related orphan receptor C (RORC), solute carrier family 26 member 6 (SLC26A6), T cell immune regulator 1, ATPase H + transporting V0 subunit a3 (TCIRG1) and X-C motif chemokine ligand 1 (XCL1) (Table 1; Additional file 3). All genes were downregulated within this set in response to gut microbiota ageing, apart from RORC which was upregulated. Moreover, the expression profile of this 25-gene signature showed a positive correlation with naïve T-cell (*P* = 8.3E−52, *R* = 0.69), effector memory T-cell (*P* = 7.3E−61, *R* = 0.73), central memory T-cell (*P* = 1.3E−41, *R* = 0.63), resident memory T-cell (*P* = 3.7E−16, *R* = 0.41), exhausted T-cell (*P* = 1.6E−27, *R* = 0.53), resting Treg T-cell (*P* = 5.1E−38, *R* = 0.61), effector Treg T-cell (*P* = 1.7E−58, *R* = 0.72), and Th1-like (*P* = 3.8E−68, *R* = 0.76), but not effector T-cell (*P* = 0.78, *R* = 0.015) gene signatures in normal colorectal tissues (Fig. 2). The Antigen Presentation Pathway was the most significantly down-regulated canonical pathway predicted by IPA (*p* < 9.3 × 10^–7^). IPA also predicted that gene expression in recipient mice transplanted with old gut microbiota was down-regulated by a decrease in cytokine signalling in response to interferon-g (*p* = 1.7 × 10^–10^), IL-1b (*p* = 2.3 × 10^–9^), IL-27 (*p* = 4.3 × 10^–8^), IL-4 (*p* = 4.5 × 10^–7^) and IL-2 (*p* = 2.2 × 10^–6^) which are key cytokines involved in the regulation of T cell function.

**Table 1:** Immune gene signatures in the protein–protein interaction network of differentially expressed genes of the small intestine from young (5–6 weeks old) germ-free mouse hosts following 8 weeks after transplantation with either old (~ 24-month old) or young (5–6 weeks old) mouse donor gut microbiota

Gene ID	Name	− log10(P)*	Log2(FC)
*Highly interacting*			
ATP7A	ATPase copper transporting alpha	3.87	− 0.46
BIRC3	Baculoviral IAP repeat containing 3	3.90	− 0.79
CASP1	Caspase 1	4.34	− 0.69
CD74	CD74 molecule	4.25	− 0.57
CXCL9	C-X-C motif chemokine ligand 9	5.33	− 1.85
ERAP1	Endoplasmic reticulum aminopeptidase 1	4.06	− 0.68
FAS	Fas cell surface death receptor	5.51	− 1.31
GBP2	Guanylate binding protein 2	4.02	− 2.03
GBP6	Guanylate binding protein family member 6	3.94	− 1.11
HLA-DMA	Major histocompatibility complex, class II, DM alpha	3.98	− 0.89
HLA-DMB	Major histocompatibility complex, class II, DM beta	3.91	− 0.66
HLA-DQB2	Major histocompatibility complex, class II, DQ beta 2	4.48	− 0.79
IDO1	Indoleamine 2,3-dioxygenase 1	5.77	− 1.89
NR1D1	Nuclear receptor subfamily 1 group D member 1	5.60	− 2.01
PSMB10	Proteasome 20S subunit beta 10	5.18	− 0.97
RORC	RAR related orphan receptor C	4.28	1.03
XCL1	X-C motif chemokine ligand 1	4.03	− 0.74
*Low interacting*			
TCIRG1	T cell immune regulator 1, ATPase H + transporting V0 subunit a3	4.00	− 0.48
*Non interacting*			
DOCK11	Dedicator of cytokinesis 11	− 0.82	− 0.82
EMP2	Epithelial membrane protein 2	− 1.00	− 1.00
FER	FER tyrosine kinase	− 0.59	− 0.59
GRN	Granulin precursor	− 1.08	− 1.08
GSDMC	Gasdermin C	− 3.35	− 3.35
GSDMC	Gasdermin C	− 4.04	− 4.04
LGR4	Leucine rich repeat containing G protein-coupled receptor 4	− 0.75	− 0.75
RNASET2	Ribonuclease T2	− 0.46	− 0.46
RNASET2	Ribonuclease T2	− 0.46	− 0.46
SLC26A6	Solute carrier family 26 member 6	− 0.69	− 0.69
DOCK11	Dedicator of cytokinesis 11	− 0.82	− 0.82
EMP2	Epithelial membrane protein 2	− 1.00	− 1.00
FER	FER tyrosine kinase	− 0.59	− 0.59
GRN	Granulin precursor	− 1.08	− 1.08
GSDMC	Gasdermin C	− 3.35	− 3.35
GSDMC	Gasdermin C	− 4.04	− 4.04
LGR4	Leucine rich repeat containing G protein-coupled receptor 4	− 0.75	− 0.75
RNASET2	Ribonuclease T2	− 0.46	− 0.46
RNASET2	Ribonuclease T2	− 0.46	− 0.46
SLC26A6	Solute carrier family 26 member 6	− 0.69	− 0.69

## Discussion

Our analysis and functional annotation of gut samples from young germ-free mice transplanted with old donor gut microbiota has identified a signature of 25 immune-associated DEGs, that were mostly downregulated following transplantation of old donor gut microbiota. These DEGs were associated with naïve T-cell, effector memory T-cell, central memory T-cell, resident memory T-cell, exhausted T-cell, resting Treg T-cell, effector Treg T-cell and Th1-like colorectal gene signatures. These genes may have a potential role as candidate markers of immune dysregulation in response to gut microbiota ageing. The analysis we report here builds upon existing research that has identified dysfunction in macrophages leading to increased cytokine production and reduced bacterial killing in older mice house in non-sterile conditions [14]. Our findings show that the adaptive immune system may also be altered by an ageing gut microbiota, with a general down-regulation of T-cell functions leading potentially to chronic inflammation.

### Major histocompatibility complex

Antigen Presentation was the most down-regulated canonical pathway in recipient mice transplanted with old gut microbiota. Antigen presentation involves the HLA system (also known as major histocompatibility complex, MHC), a complex of genes encoding cell-surface proteins that regulate immune function by presenting processed peptide antigens [15]. MHC-encoded HLA genes (i.e., HLA-DQB2, HLA-DMB, HLA-DMA) have been shown to alter microbial composition [16], particularly in resistant-HLA-DR transgenic mice that demonstrate enriched *Porphyromonadaceae* and *Bifidobacteria* species [17]. Likewise, research in humans with HLA-DR genotype has revealed a lower microbial diversity which is associated with a greater risk of developing coeliac disease [18], as well as ankylosing spondylitis and rheumatoid arthritis [19]. MHC II is a complex of protein subunits involved in antigen presentation to T cells and which is responsible for the development of an adaptive immune response to infection [20]. CD74 is a subunit of MHC Class II and is expressed in intestinal and gastric epithelial cells [21]. Low expression of CD74 may compromise antigen presentation in the gut, leading to subsequent impaired immune system in response to gut dysbiosis [22]. The major source of intestinal MHC Class II expression is likely to be gut macrophages. These innate immune cells have previously been identified as having an altered phenotype in older mice, driven by microbial dysbiosis, leading to increased cytokine production but lower bacterial killing capacity [14]. ERAP1 plays a key role in the cleavage of antigens in the endoplasmic reticulum for expression on MHC I molecules [23, 24]. ERAP1 may modulate innate immunity as observed in mice lacking this protein which demonstrate higher inflammation levels [25]. Additionally, EPAP1 may aid in nitric oxide synthesis [26] and facilitate in the shedding of IL-6, TNF-a, and IL-1 cytokine receptors from the plasma membrane [27]. Evidence has suggested that ERAP1 variants have an established role in autoimmune disorders, which has been described in ERAP1 deficient mice that exhibit reduced T and dendritic cell count, linked with ankylosing spondylitis [28, 29]. Interestingly, the gut microbiota has been proposed as potential mechanisms in driving the pathogenesis of ankylosing spondylitis, highlighting the immunomodulatory influence of a perturbed microbiome [30].

### Chemokines, cytokines, and interferons

Our analysis of DEGs from recipient mice transplanted with old gut microbiota also predicted a down-regulation of genes in response to a decrease in expression of key cytokines (IFNg, IL-1b, IL-27, IL-4, IL-2) involved in T cell activation and differentiation. Cytokines and chemokines have been proposed as potential mediators of intestinal barrier that may protect against microbiota-induced inflammation [31]. XCL1 is a chemokine that has been proposed as a regulator of intestinal homeostasis. XCL-1-deficient mice have dysregulated gut milieu, characterised by increased Th1/Th17 cells and decreased Treg cells [32]. Mice with diminished intestinal T cells and accumulated dendritic cells in the gut upon have also been described upon XCL-1 deficiency [33]. CXCL9 is another key chemokine which may recruit T cells by binding to its CXCR3 receptor [34] and inhibit intestinal cell apoptosis [35], as shown in inflated and infected gut models [36]. Specifically, CXCL9 has been associated with regenerated islet-derived protein-mediated microbiota expression, which when under-expressed is suggested to be responsible for lower microbial diversity [37]. Moreover, DOCK11, a mediator of cytokinesis, is another contributor of immunosenescence during ageing, although independent of B cell antibody responses [38]. For instance, mice with DOCK11-deificient B cells have reduced antigen-specific participation in germinal centers, which is accompanied by lower B cell intrinsic-signaling stimulation [39]. Furthermore, the interleukin-1 converting enzyme CASP1, is a protease which converts the inactive form of IL-1Β to its active form, that is a precursor of inflammatory processes [40]. CASP1 ablation in intestinal epithelial cells of mice has a protective response against inflammation-induced intestinal tumors compared to controls, independent of gut microbiota composition [41], suggesting an immune-deprived status under normal physiology upon downregulation. Finally, profiles of enhanced systemic inflammation have been observed following fecal microbiota transplantation of CASP1-deficient mice into Ldlr-deficient mice, further augmenting the impact of CASP1 on gut permeability disruption [42]. In addition, guanylate binding proteins (GBPs) are a family of GTPases that are induced by interferon signalling and which drive inflammation. Interferons are key mediators of the cellular immune system defense against infected host cells, as previously observed in GBP (GBP2 and GBP6) knock-out mice [43]. GBPs may also promote phagocyte oxidase, antimicrobial peptides, and autophagy effectors to provide protection against inflammation [44] and intracellular pathogens, including *L. monocytogenes, Francisella novicida*, and Mycobacteria [45–47]. PSMB10 is an immunoproteasome gene which is stimulated by interferon-γ (IFNγ) [48]. *Toxoplasma gondii* infected mice have exhibited an increased expression of PSMB10 in both the RNA and protein levels compared to uninfected controls (French et al. 2021). Infection with *Toxoplasma gondii* induces the secretion of proinflammatory cytokines, including TNF-a [49]. PSMB10 has been considered a risk factor for the regulation of viral response and epithelial cell differentiation, particularly during immunocompromised-induced conditions such as cancer [19].

### Membrane proteins

Seven down-regulated DEGs in recipient mice transplanted with old gut microbiota can be classified as membrane proteins (EMP2, GRN, SLC26A6, LGR4, TCIRG, ATP7A, FER). EMP2 has been shown to confer a potential therapeutic role against host infection [50]. In particular, increased EMP2 expression promotes integrin α6β1 and αVβ3 expression, which facilitates immune cell trafficking, effector cell activation, and proliferation in tissues [51]. EMP2 deficiency in mice has been linked with impaired epithelial neutrophil migration [52], while a strong transcriptomic down-regulation of EMP2 has been observed in inflammatory lung disease [53]. GRN, an immune-regulatory molecule, has been shown to modulate TNF/TNFR signaling via increased leukocyte elastase activity, which becomes triggered by neutrophil stimulation [54]. Indeed, increased mucosal progranulin (PGRN) expression in gastric epithelial cells following infection with *Helicobacter pylori* has been observed [55, 56]. Similarly, research in vivo has demonstrated that PGRN-deficient mice are unable to eliminate pathogenic bacteria compared to wild type mice [57]. These results indicate that GRN has an immunomodulatory role with a potential to alleviate inflammatory-induced responses from bacterial infection [58]. SLC26A6 is a chloride-oxalate exchanger that plays a key role in oxalate secretion in the gut, regulated by *Oxalobacter formigenes* [59]. Research has shown that antibiotic administration may induce repercussions to the gut microbiota, impairing oxalate transport and metabolism [59]. Mice lacking SLC26A6 have impaired intestinal oxalate secretion [60, 61], which disrupts NLRP3 inflammasome sensors to mediate gut microbiota in inhibiting leakage of toll-like receptor agonists. This may lead to IL-1Β secretion inducing pyroptosis [62]. LGR4, a member of leucine-rich repeat-containing G protein-coupled receptors (LGRs), is a mediator of immune response via macrophage activity. LGR4 knock-out mice have exhibited a pro-inflammatory transcriptional signature [63], whereas peripheral blood mononuclear cells of antibiotic-treated monkeys have revealed a prominent role of Lgr4 in immune homeostasis compared to germ-free monkeys [64]. TCIRG1 is a gene encoding a subunit of vacuolar V-ATPase that has been shown to be highly expressed in gut endothelial cells after *Salmonella typhimurium* infection [65]. The TIRC7 isoform is expressed on the membrane of T cells and is essential for normal T cell function. Although there are limited data on its direct link with the gut microbiota, weaned piglets fed with *Lactobacillus gasseri LA39* exhibit significantly increased TCIRG1 expression and cellular ATP levels in intestinal epithelial cells [66]. In addition, ATP7A is considered a gene promoting the regulation of copper concentrations throughout the body, which has been found to be significantly downregulated in the colon of antibiotic-treated mice compared to conventionally raised controls [67]. ATP7A has been linked with attenuated bacterial elimination, that may be explained by reduced macrophage bactericidal activity [68, 69].

FER is a non-transmembrane receptor cytosolic tyrosine kinase that acts down-stream of membrane receptors to regulate the activation of inflammatory monocytes and macrophages [70]. Interestingly, absence of FER has demonstrated an exacerbated recruitment of leukocytes in response to lipopolysaccharide induction in FER-mutant relative to wild type mice, highlighting its critical role in innate immune response [71]. In this context, FER-electroporated C57/BL6 mice have shown an increased phosphorylation of IL-1β, Nrf2, Nlrp3, Cxcl2, and HSP90, and a higher stimulation of TNF-α, CCL-2, KC, IFN-γ and IL-1RA [72]. In addition, FER overexpression has demonstrated enhanced innate immunity and bacterial clearance amongst mice models [72, 73].

### Transcription factors

Several transcription factors were differentially expressed in mice transplanted with old gut microbiota. Nuclear Receptor Subfamily 1 Group D Member 1 (NR1D1) is a transcription factor with a pivotal role on core circadian regulation [74] and processes under circadian homeostasis, including pathways of the immune system [75]. High NR1D1 levels are implicated in increased and sustained corticosterone secretion through reduced gene encoding nuclear factor interleukin-3-regulated protein (Nfil3) expression in germ-free mice compared to those with a normal gut microbiota. These changes may be established via toll-like receptors (TLRs) affecting arrhythmic bacterial signaling and disrupting circadian rhythms [76, 77]. RORC is gene encoding of the transcription factor RAR-related orphan-like γt (RORγt) that plays a critical role in regulation of innate ILC3 and Th17 cells [78]. For instance, RORγt innate ILC3 cells may be used by the commensal microbes to upregulate T cell function and prevent aberrant inflammatory responses, linked with dysregulated Th17 cells and inflammatory bowel disease (IBD) in mice models [79, 80]. Likewise, microbial species from humans with IBD may alter Th17 cell balance and ROR(γt) regulatory T cells in vivo [81], displaying further the relationship between dysregulated immune function and intestinal inflammation. RORC was the only DEG that was upregulated in recipient mice transplanted with old gut microbiota.

### Toll-like receptor pathway

BIRC3 is a host gene involved in inhibiting pathways of apoptosis and autophagy processes [82, 83]. Its overexpression may be achieved via the TLR4/NFκB pathway activation that initiates NLRP3 agonists to stimulate inflammatory responses and IL-1β and IL-18 secretion, leading to Th17 cell differentiation and mitophagy elimination [84, 85]. RNASET2 is a ribonuclease cleaving or degrading RNA molecules, imposing a vital role on the mediation of inflammatory states [86]. The possessed regulatory role of RNASET2 may be accomplished through TLR8 activation that induces Th1 cell response to shield against intracellular pathogens [87]. RNASET2 has been correlated with Graves’ disease, an autoimmune disorder featuring hyperthyroidism, as revealed in a previous genome-wide association study [88].

### Amino acid metabolism

IDO1 is an enzyme enabling the generation of tryptophan derivatives (i.e., kynurenines), which are identified as aryl hydrocarbon receptor (AhR) ligands [89]. These tryptophan metabolites may be produced by various *Lactobacilli* species in a synbiotic human microbiome, thereby inducing anti-inflammatory properties, protecting immune balance [89]. Particularly, IDO1 is suggested to have immunomodulatory effects acting upon dendritic cells for the prevention of inflammatory states [90], where its phosphorylation may promote an endotoxin-tolerant state, protecting against infections as seen previously [91]. Additionally, supplementation of Lactobacillus-containing probiotic in HIV-infected macaques models revealed a reduced IDO1 activity, which may help maintaining Th17 cells. IDO1 expression in intestinal epithelial cells of IDO1-positive mice promotes mucus production, increased populations of *Akkermansia muciniphila* and *Mucispirillum schaedleri* that corresponds with higher secretory cell differentiation levels [92]. The aforementioned outcomes further illustrate the importance of Lactobacillus-based bacteria as regulators of immune homeostasis in the intestinal mucosa [93].

## Conclusions and future work

Diversity and compositional changes in the gut microbiota are linked with perturbed immune homeostasis during ageing. Genetic insights into the interaction between age-related changes in gut microbiota and immune function, remain ill-defined. We identified a 25-gene signature of immune-associated DEGs in the small intestine of young germ-free mice transplanted with old donor gut microbiota using publicly available datasets. These genes may have a potential role as markers of immune dysregulation in the ageing of the gut microbiota. Moreover, these DEGs provide insights into the altered immune response to microbiota in the ageing gut, including reduced antigen presentation and alterations in cytokine and chemokine production. Future experimental work should focus on validation of the data reported here, including that validation of innate and adaptive immune cell populations and phenotypes within the gut of younger and older mice, and the changes in immune cell populations and cellular functions associated with microbial dysbiosis during ageing.

## Supplementary Information

**Additional file 1.** Volcano plot of differentially expressed genes (DEGs) of the small intestine from young (5-6 weeks old) germ-free mouse hosts following 8 weeks after transplantation with either old (~24-month old) or young (5-6 weeks old) mouse donor gut microbiota. DEGs with an adjusted P < 0.05 (corrected by the Benjamini-Hochberg False Discovery Rate) were considered as significant.

**Additional file 2.** Differentially expressed genes of the small intestine from young (5-6 weeks old) germ-free mouse hosts following 8 weeks after transplantation with either old (~24-month old) or young (5-6 weeks old) mouse donor gut microbiota.

**Additional file 3.** Functional enrichment of differentially expressed genes from the small intestine of young recipient mice transplanted with either old donor gut microbiota or young donor counterparts. The top 10 gene ontology annotations based on biological process are shown.

## Data Availability

The datasets analysed during the current study are available in the NCBI Gene Expression Omnibus repository (https://www.ncbi.nlm.nih.gov/geo/query/acc.cgi?acc=GSE130026).

## Abbreviations

ATP7A: ATPase copper transporting alpha
BIRC3: Baculoviral IAP repeat containing 3
BP: Biological process
CASP1: Caspase 1
CD74: CD74 molecule
CXCL9: C-X-C motif chemokine ligand 9
DEGs: Differentially expressed genes
DOCK11: Dedicator of cytokinesis 11
EMP2: Epithelial membrane protein 2
ERAP1: Endoplasmic reticulum aminopeptidase 1
FAS: Fas cell surface death receptor
FER: FER tyrosine kinase
GBP2: Guanylate binding protein 2
GBP6: Guanylate binding protein 2
GEO: Gene expression omnibus
GEPIA2: Gene expression profiling interactive analysis 2
GO: Gene ontology
GRN: Granulin precursor
GTEx: Genotype-tissue expression
HLA-DMA: Major histocompatibility complex, class II, DM alpha
HLA-DMB: Major histocompatibility complex, class II, DM beta
HLA-DQB2: Major histocompatibility complex, class II, DQ beta 2
IDO1: Indoleamine 2,3-dioxygenase 1
IPA: Ingenuity pathway analysis
LGR4: Leucine rich repeat containing G protein-coupled receptor 4
MSigDB: Molecular signatures database
NCBI: National Center for Biotechnology Information
NLM: National Library of Medicine
NR1D1: Nuclear receptor subfamily 1 group D member 1
PPI: Protein–protein interactions
PSMB10: Proteasome 20S subunit beta 10
RNASET2: Ribonuclease T2
RORC: RAR related orphan receptor C
SLC26A6: Solute carrier family 26 member 6
STRING: The search tool for the retrieval of interacting genes
TCGA: The Cancer Genome Atlas
TCIRG1: T cell immune regulator 1, ATPase H + transporting V0 subunit a3
XCL1: X-C motif chemokine ligand
