# Supplementary figures and images for: Markers of immune dysregulation in response to the ageing gut: insights from aged murine gut microbiota transplants
Source: BMC Gastroenterol. 2022 Dec 21;22:533. doi: 10.1186/s12876-022-02613-2 (PMC9773626; doi:10.1186/s12876-022-02613-2)

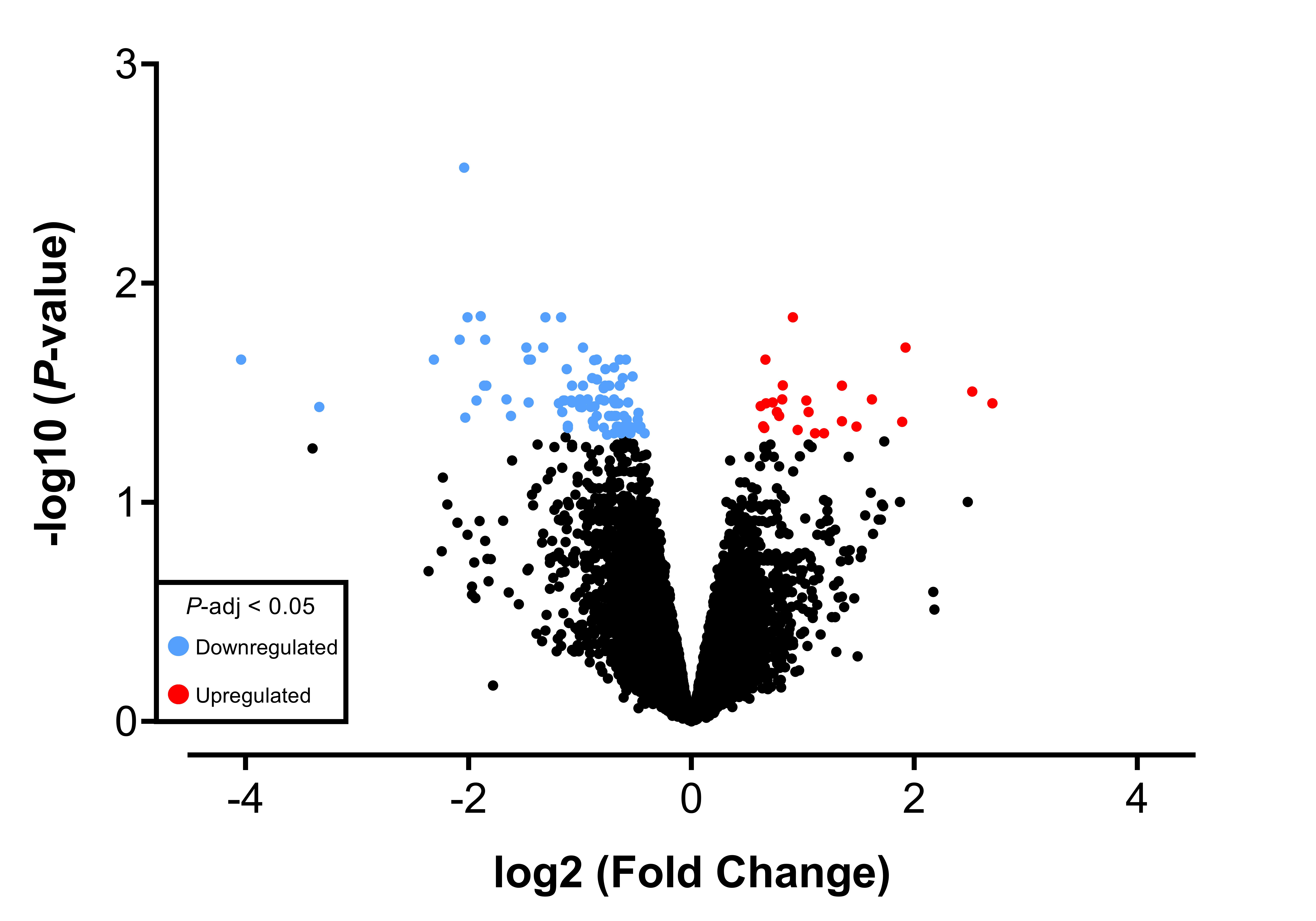

Supplement: Supplementary file 1 — Additional file 1. Volcano plot of differentially expressed genes (DEGs) of the small intestine from young (5-6 weeks old) germ-free mouse hosts following 8 weeks after transplantation with either old (~24-month old) or young (5-6 weeks old) mouse donor gut microbiota. DEGs with an adjusted P < 0.05 (corrected by the Benjamini-Hochberg False Discovery Rate) were considered as significant. [file 12876_2022_2613_MOESM1_ESM.jpg]
