# Supplementary material for: Markers of immune dysregulation in response to the ageing gut: insights from aged murine gut microbiota transplants
Source: BMC Gastroenterol. 2022 Dec 21;22:533. doi: 10.1186/s12876-022-02613-2 (PMC9773626; doi:10.1186/s12876-022-02613-2)
Supplement: Supplementary file 2 — Additional file 2. Differentially expressed genes of the small intestine from young (5-6 weeks old) germ-free mouse hosts following 8 weeks after transplantation with either old (~24-month old) or young (5-6 weeks old) mouse donor gut microbiota. [file 12876_2022_2613_MOESM2_ESM.docx]

| **Gene ID** | **LogFC** | **P-value** | **Gene Title** |
| --- | --- | --- | --- |
| *Upregulated* | |  |  |
| NMT1 | 1.12 | 1.48E-04 | N-myristoyltransferase 1 |
| PER2 | 1.35 | 3.09E-05 | period circadian regulator 2 |
| PPIL6 | 0.62 | 6.61E-05 | peptidylprolyl isomerase like 6 |
| RND2 | 0.65 | 1.17E-04 | Rho family GTPase 2 |
| SLC47A1 | 1.35 | 1.05E-04 | solute carrier family 47 member 1 |
| ODC1 | 0.79 | 8.97E-05 | ornithine decarboxylase 1 |
| FLVCR2 | 1.62 | 4.58E-05 | FLVCR heme transporter 2 |
| EPHX2 | 1.48 | 1.21E-04 | epoxide hydrolase 2 |
| GPT | 0.81 | 4.44E-05 | glutamic--pyruvic transaminase |
| ANGPTL4 | 1.89 | 1.07E-04 | angiopoietin like 4 |
| ACAA2 | 1.19 | 1.50E-04 | acetyl-CoA acyltransferase 2 |
| CRY2 | 0.66 | 1.11E-05 | cryptochrome circadian regulator 2 |
| S100A6 | 0.77 | 7.78E-05 | S100 calcium binding protein A6 |
| RORC | 1.03 | 5.21E-05 | RAR related orphan receptor C |
| PDK4 | 2.52 | 3.62E-05 | pyruvate dehydrogenase kinase 4 |
| FCHSD2 | 0.91 | 3.07E-06 | FCH and double SH3 domains 2 |
| PEX11A | 0.95 | 1.37E-04 | peroxisomal biogenesis factor 11 alpha |
| RAB4A | 0.67 | 6.32E-05 | RAB4A, member RAS oncogene family |
| NEK3 | 0.66 | 1.31E-04 | NIMA related kinase 3 |
| NT5E | 2.70 | 6.14E-05 | 5'-nucleotidase ecto |
| RPSA | 1.92 | 5.71E-06 | ribosomal protein SA |
| SPA17 | 0.82 | 2.41E-05 | sperm autoantigenic protein 17 |
| DIPK2A | 1.05 | 7.80E-05 | divergent protein kinase domain 2A |
| TSC22D3 | 0.73 | 5.66E-05 | TSC22 domain family member 3 |
|  |  |  |  |
| *Downregulated* | |  |  |
| PM20D1 | -1.17 | 2.13E-06 | peptidase M20 domain containing 1 |
| MFSD4A | -0.78 | 5.08E-05 | major facilitator superfamily domain containing 4A |
| XCL1 | -0.74 | 9.35E-05 | X-C motif chemokine ligand 1 |
| SMPDL3A | -1.33 | 7.13E-06 | sphingomyelin phosphodiesterase acid like 3A |
| HKDC1 | -0.69 | 8.53E-05 | hexokinase domain containing 1 |
| GRN | -1.08 | 5.21E-05 | granulin precursor |
| SCPEP1 | -1.44 | 1.22E-05 | serine carboxypeptidase 1 |
| B4GALNT2 | -0.77 | 2.96E-05 | beta-1,4-N-acetyl-galactosaminyltransferase 2 |
| NR1D1 | -2.01 | 2.54E-06 | nuclear receptor subfamily 1 group D member 1 |
| TNS4 | -2.08 | 4.82E-06 | tensin 4 |
| TGFBI | -1.14 | 4.99E-05 | transforming growth factor beta induced |
| ERAP1 | -0.68 | 8.63E-05 | endoplasmic reticulum aminopeptidase 1 |
| TMEM171 | -1.15 | 4.97E-05 | transmembrane protein 171 |
| CTSB | -0.70 | 1.50E-04 | cathepsin B |
| CCDC198 | -1.93 | 5.18E-05 | coiled-coil domain containing 198 |
| ZDHHC20 | -0.77 | 1.65E-05 | zinc finger DHHC-type palmitoyltransferase 20 |
| CPQ | -1.16 | 7.87E-05 | carboxypeptidase Q |
| ATP6V1C1 | -0.52 | 1.30E-04 | ATPase H+ transporting V1 subunit C1 |
| TMEM117 | -0.76 | 1.56E-04 | transmembrane protein 117 |
| C5orf51 | -0.42 | 1.48E-04 | chromosome 5 open reading frame 51 |
| GSDMC | -3.35 | 7.03E-05 | gasdermin C |
| GSDMC | -4.04 | 1.23E-05 | gasdermin C |
| PARP10 | -0.87 | 1.36E-05 | poly(ADP-ribose) polymerase family member 10 |
| SLC39A4 | -0.85 | 9.06E-05 | solute carrier family 39 member 4 |
| KLHL24 | -1.11 | 1.29E-04 | kelch like family member 24 |
| CSTA | -2.04 | 2.16E-07 | cystatin A |
| ZBTB20 | -0.55 | 1.51E-04 | zinc finger and BTB domain containing 20 |
| EMP2 | -1.00 | 4.17E-05 | epithelial membrane protein 2 |
| ADGRG7 | -0.67 | 6.28E-05 | adhesion G protein-coupled receptor G7 |
| RNASET2 | -0.46 | 1.19E-04 | ribonuclease T2 |
| ABCA3 | -0.55 | 1.25E-04 | ATP binding cassette subfamily A member 3 |
| PPARD | -1.12 | 1.71E-05 | peroxisome proliferator activated receptor delta |
| PIM1 | -1.07 | 2.88E-05 | Pim-1 proto-oncogene, serine/threonine kinase |
| HLA-DMA | -0.89 | 1.05E-04 | major histocompatibility complex, class II, DM alpha |
| HLA-DMB | -0.66 | 1.23E-04 | major histocompatibility complex, class II, DM beta |
| PSMB8 | -0.89 | 2.09E-05 | proteasome 20S subunit beta 8 |
| HLA-DQB2 | -0.79 | 3.33E-05 | major histocompatibility complex, class II, DQ beta 2 |
| FER | -0.59 | 1.09E-05 | FER tyrosine kinase |
| RNASET2 | -0.46 | 1.32E-04 | ribonuclease T2 |
| CD74 | -0.57 | 5.66E-05 | CD74 molecule |
| ABHD3 | -0.88 | 1.17E-04 | abhydrolase domain containing 3, phospholipase |
| CORO1B | -0.62 | 2.06E-05 | coronin 1B |
| STAMBPL1 | -0.74 | 2.62E-05 | STAM binding protein like 1 |
| FAS | -1.31 | 3.11E-06 | Fas cell surface death receptor |
| AS3MT | -0.85 | 2.19E-05 | arsenite methyltransferase |
| TCIRG1 | -0.48 | 1.01E-04 | T cell immune regulator 1, ATPase H+ transporting V0 subunit a3 |
| VPS51 | -1.00 | 7.01E-05 | VPS51 subunit of GARP complex |
| MS4A12 | -0.72 | 8.93E-05 | membrane spanning 4-domains A12 |
| FUBP3 | -0.53 | 1.93E-05 | far upstream element binding protein 3 |
| UBR3 | -0.58 | 1.01E-04 | ubiquitin protein ligase E3 component n-recognin 3 |
| LGR4 | -0.75 | 2.98E-05 | leucine rich repeat containing G protein-coupled receptor 4 |
| DPP7 | -0.61 | 9.16E-05 | dipeptidyl peptidase 7 |
| GPR160 | -1.07 | 5.51E-05 | G protein-coupled receptor 160 |
| MFSD1 | -0.85 | 1.24E-05 | major facilitator superfamily domain containing 1 |
| EIF4E | -0.69 | 1.52E-05 | eukaryotic translation initiation factor 4E |
| GBP2 | -2.03 | 9.62E-05 | guanylate binding protein 2 |
| WLS | -1.62 | 8.77E-05 | Wnt ligand secretion mediator |
| TMEM144 | -0.70 | 5.19E-05 | transmembrane protein 144 |
| TMCO4 | -0.48 | 8.04E-05 | transmembrane and coiled-coil domains 4 |
| BPNT2 | -0.55 | 1.52E-04 | 3'(2'), 5'-bisphosphate nucleotidase 2 |
| TTC39B | -0.79 | 3.42E-05 | tetratricopeptide repeat domain 39B |
| DIO1 | -2.31 | 1.14E-05 | iodothyronine deiodinase 1 |
| P2RX4 | -0.61 | 1.21E-04 | purinergic receptor P2X 4 |
| LETM1 | -0.64 | 3.04E-05 | leucine zipper and EF-hand containing transmembrane protein 1 |
| SLC2A9 | -0.67 | 1.22E-04 | solute carrier family 2 member 9 |
| CXCL9 | -1.85 | 4.71E-06 | C-X-C motif chemokine ligand 9 |
| GBP6 | -1.11 | 1.14E-04 | guanylate binding protein family member 6 |
| DIPK1A | -0.93 | 4.28E-05 | divergent protein kinase domain 1A |
| OCM2 | -0.97 | 3.20E-05 | oncomodulin 2 |
| ATP6V0E2 | -0.90 | 7.10E-05 | ATPase H+ transporting V0 subunit e2 |
| PON3 | -1.19 | 6.01E-05 | paraoxonase 3 |
| KLK1 | -1.67 | 4.49E-05 | kallikrein 1 |
| CASP3 | -1.48 | 6.79E-06 | caspase 3 |
| CPNE2 | -1.84 | 3.03E-05 | copine 2 |
| IDO1 | -1.89 | 1.71E-06 | indoleamine 2,3-dioxygenase 1 |
| DCTN6 | -0.65 | 6.03E-05 | dynactin subunit 6 |
| SLC25A4 | -1.86 | 3.16E-05 | solute carrier family 25 member 4 |
| PSMB10 | -0.97 | 6.61E-06 | proteasome 20S subunit beta 10 |
| MLKL | -1.46 | 9.10E-06 | mixed lineage kinase domain like pseudokinase |
| CASP1 | -0.69 | 4.59E-05 | caspase 1 |
| VWA5A | -0.98 | 7.19E-05 | von Willebrand factor A domain containing 5A |
| SLC26A6 | -0.69 | 6.18E-05 | solute carrier family 26 member 6 |
| BIRC3 | -0.79 | 1.27E-04 | baculoviral IAP repeat containing 3 |
| DOCK11 | -0.82 | 4.18E-05 | dedicator of cytokinesis 11 |
| NSDHL | -1.46 | 5.55E-05 | NAD(P) dependent steroid dehydrogenase-like |
| CHIC1 | -0.64 | 1.23E-05 | cysteine rich hydrophobic domain 1 |
| ATP7A | -0.46 | 1.35E-04 | ATPase copper transporting alpha |
| SH3BGRL | -0.68 | 5.75E-05 | SH3 domain binding glutamate rich protein like |
| ACOT9 | -0.63 | 1.52E-04 | acyl-CoA thioesterase 9 |
| MOSPD2 | -0.87 | 6.71E-05 | motile sperm domain containing 2 |
