# Supplementary material for: Markers of immune dysregulation in response to the ageing gut: insights from aged murine gut microbiota transplants
Source: BMC Gastroenterol. 2022 Dec 21;22:533. doi: 10.1186/s12876-022-02613-2 (PMC9773626; doi:10.1186/s12876-022-02613-2)
Supplement: Supplementary file 3 — Additional file 3. Functional enrichment of differentially expressed genes from the small intestine of young recipient mice transplanted with either old donor gut microbiota or young donor counterparts. The top 10 gene ontology annotations based on biological process are shown. [file 12876_2022_2613_MOESM3_ESM.docx]

| **GO Term** | **Description** | **Genes** | **P-value** |
| --- | --- | --- | --- |
| Small molecule metabolic process | The chemical reactions and pathways involving small molecules, any low molecular weight, monomeric, non-encoded molecule. | 30 | 4.03E-12 |
| Homeostatic process | Any biological process involved in the maintenance of an internal steady state. | 28 | 2.17E-10 |
| Transmembrane transport | The process in which a solute is transported across a lipid bilayer, from one side of a membrane to the other. | 24 | 9.17E-09 |
| Organic acid metabolic process | The chemical reactions and pathways involving organic acids, any acidic compound containing carbon in covalent linkage. | 19 | 3.58E-08 |
| Immune response | Any immune system process that functions in the calibrated response of an organism to a potential internal or invasive threat. | 25 | 6.51E-08 |
| Chemical homeostasis | Any biological process involved in the maintenance of an internal steady state of a chemical. | 20 | 1.45E-07 |
| Biological process involved in interspecies interaction between organisms | Any process evolved to enable an interaction with an organism of a different species. | 22 | 7.65E-07 |
| Programmed cell death | A process which begins when a cell receives an internal or external signal and activates a series of biochemical events (signaling pathway). The process ends with the death of the cell. | 23 | 2.72E-06 |
| Lipid metabolic process | The chemical reactions and pathways involving lipids, compounds soluble in an organic solvent but not, or sparingly, in an aqueous solvent. Includes fatty acids; neutral fats, other fatty-acid esters, and soaps; long-chain (fatty) alcohols and waxes; sphingoids and other long-chain bases; glycolipids, phospholipids and sphingolipids; and carotenes, polyprenols, sterols, terpenes and other isoprenoids. | 19 | 6.64E-06 |
| Ion transport | The directed movement of charged atoms or small charged molecules into, out of or within a cell, or between cells, by means of some agent such as a transporter or pore. | 20 | 6.64E-06 |
